# Balance, gait, and navigation performance are related to physical exercise in blind and visually impaired children and adolescents

**DOI:** 10.1007/s00221-021-06038-3

**Published:** 2021-02-07

**Authors:** Ann-Kathrin Rogge, Daniel Hamacher, Giulia Cappagli, Laura Kuhne, Kirsten Hötting, Astrid Zech, Monica Gori, Brigitte Röder

**Affiliations:** 1 Biological Psychology and Neuropsychology, University of Hamburg, Von-Melle-Park 11, 20146 Hamburg, Germany; 2 Max Planck School of Cognition, Max-Planck Institute for Human Cognitive and Brain Sciences, Leipzig, Germany; 3 Institute of Sport Science, Friedrich Schiller University, Jena, Germany; 4 Unit for Visually Impaired People, Istituto Italiano di Tecnologia, U-VIP, Genoa, Italy; 5 Friedrich Schiller University, Statistics and Methods in Sports, Jena, Germany

**Keywords:** Postural control, Visual deprivation, Physical activity, Spatial cognition, Vestibular system

## Abstract

Self-motion perception used for locomotion and navigation requires the integration of visual, vestibular, and proprioceptive input. In the absence of vision, postural stability and locomotor tasks become more difficult. Previous research has suggested that in visually deprived children, postural stability and levels of physical activity are overall lower than in sighted controls. Here we hypothesized that visually impaired and blind children and adolescents differ from sighted controls in postural stability and gait parameters, and that physically active individuals outperform sedentary peers in postural stability and gait parameters as well as in navigation performance. Fourteen blind and visually impaired children and adolescents (8–18 years of age) and 14 matched sighted individuals took part. Assessments included postural sway, single-leg stance time, parameters of gait variability and stability, self-reported physical activity, and navigation performance. Postural sway was larger and single-leg stance time was lower in blind and visually impaired participants than in blindfolded sighted individuals. Physical activity was higher in the sighted group. No differences between the group of blind and visually impaired and blindfolded sighted participants were observed for gait parameters and navigation performance. Higher levels of physical activity were related to lower postural sway, longer single-leg stance time, higher gait stability, and superior navigation performance in blind and visually impaired participants. The present data suggest that physical activity may enhance postural stability and gait parameters, and thereby promote navigation performance in blind and visually impaired children and adolescents.

**Supplementary Information:**

The online version contains supplementary material available at 10.1007/s00221-021-06038-3.

## Introduction

To estimate self-motion used for upright standing, locomotion and navigation, the brain integrates incoming sensory signals from visual, vestibular, and proprioceptive senses (Cullen and Taube 2017). Self-motion perception becomes less precise when visual input is temporally eliminated such as when the eyes are closed (Hartmann et al. 2013). In case of permanent blindness, larger postural sway during upright standing has been reported in blind children and adults compared to sighted controls with eyes open, indicative of postural instability (Müürsepp et al. 2018). Postural control deficits have also been found in visually impaired children with amblyopia and strabismus (Zipori et al. 2018), suggesting that a moderate visual impairment is sufficient to interfere with postural stability. During walking, slower gait velocity, shorter stride length, limited ankle plantar flexion, and a prolonged duration of stance in blind children and adults compared to sighted control participants have been reported (Bennett et al. 2019; Hallemans et al. 2010, 2011; Nakamura 1997). The altered gait patterns in the absence of vision have been interpreted as a more cautious walking strategy (Hallemans et al. 2011; Nakamura 1997). Notably, when sighted individuals were tested with closed eyes, deficits in balance as well as gait variability have been described to increase to similar levels as those of the blind (Campayo-Piernas et al. 2017; da Silva et al. 2018; Duarte and Zatsiorsky 2002; Hallemans et al. 2010; Müürsepp et al. 2018; Schmid et al. 2011; Schwesig et al. 2011; Wuehr et al. 2013). These results indicate a major role of vision for motor control during upright standing and bipedal locomotion, irrespective of whether visual input had been permanently or temporally absent.

Vision plays a pivotal role in orientation and wayfinding, too. To understand the spatial properties of an environment and to continuously update one’s own position within the environment, visual (e.g., optic flow and visual landmarks), auditory, vestibular, proprioceptive, and motor signals are integrated to update mental spatial representations (Loomis et al. 1993, 2001; Medendorp and Selen 2017; Schinazi et al. 2016). Navigation and wayfinding without vision is a particular challenge because precise spatial information from distal cues are not assessable (Loomis et al. 1993; Schinazi et al. 2016). In the dark, navigation is primarily accomplished via path integration, during which the estimates of direction, distance travelled as well as velocity are derived from vestibular and proprioceptive cues (Allen et al. 2004; Cullen and Taube 2017; Medendorp and Selen 2017). In route navigation tasks, in which participants have to reproduce a previously walked and memorized route, hardly any performance differences between blind and sighted adults have been found (Loomis et al. 1993; Rieser et al. 1986; Seemungal et al. 2007; Thinus-Blanc and Gaunet 1997). By contrast, inconsistent results exist for inferential path integration, in which participants have to deduct new multi-segment routes based on previously experienced spatial relationships such as the completion of a triangle: Blind compared to sighted individuals have been described to perform better, similar, and worse in path completion tasks (Loomis et al. 1993; Rieser et al. 1986; Seemungal et al. 2007; Thinus-Blanc and Gaunet 1997; Tinti et al. 2006). Several explanations for the inconsistent results have been suggested, including the heterogeneity of blind samples with regard to age and onset of blindness, small sample sizes, and individual differences with respect to efficient navigation strategies as well as past experience with wayfinding (Schinazi et al. 2016). For example, it has been suggested that the large inter-individual differences in path integration performance in blind individuals might be related to their habitual physical activity (Seemungal et al. 2007). The authors observed that blind individuals who performed above average in path integration tasks and displayed ultra-short vestibular time constants were those who reported higher physical activity scores. Thus, more experience with locomotor tasks in physically active individuals might be related to better wayfinding and orientation skills.

There is converging evidence in sighted humans showing a beneficial role of physical activity on cognitive functions, and specifically on memory and spatial cognition (Cassilhas et al. 2016; Fernandes et al. 2017; Stimpson et al. 2018). It has been suggested that the vestibular system might play a crucial role in mediating the link between physical activity and neural changes in cortical and subcortical brain areas including the medial temporal lobe, which have been associated with memory functions and spatial cognition (for a review see Smith 2017). The vestibular system has not only been found to be crucially involved in postural control such as during balancing on unstable ground (Lucieer et al. 2018), but several studies have additionally suggested a significant vestibular contribution to spatial cognitive functions (Angelaki et al. 2009; Hitier et al. 2014; Seemungal 2015). For example, galvanic vestibular stimulation was shown to influence performance in a visual-spatial task (Lenggenhager et al. 2007). In a longitudinal study, we have recently shown that balance training was capable of improving spatial cognitive functions such as mental rotation and perspective-taking in sighted adults (Rogge et al. 2017). Moreover, in this study, we found gray matter changes in visual and vestibular cortical areas associated with self-motion perception and spatial cognitive functions (Rogge et al. 2018). These patterns are in line with results from cross-sectional studies reporting a link between balance performance and navigation skills, mental rotation abilities, and visuo-spatial working memory in sighted adults and children as young as 6 years (Dordevic et al. 2018; Frick and Möhring 2015; Jansen and Heil 2010).

Visually impaired and blind adults as well as children have been reported to more often adopt a sedentary lifestyle, compared to their sighted peers (Augestad and Jiang 2015; Houwen et al. 2009; Longmuir and Bar-Or 2000; Müürsepp et al. 2018). However, physically active blind children and adults were found to outperform blind sedentary individuals in postural tasks and displayed enhanced gait velocity (Aydoğ et al. 2006; da Silva et al. 2018; Müürsepp et al. 2018). A recent study in blind adults has shown that 12 weeks of balance training was sufficient to increase blind participants' balance performance to a level of untrained sighted adults with eyes open (Rogge et al. 2019). By contrast, cardiorespiratory fitness had not changed after training, suggesting a specific gain in postural control and self-motion perception which could not be explained by an increase in overall physical fitness.

Yet it is unknown to which degree balance control, gait, and physical activities are related to navigation performance in blind children and whether the level of physical activity might explain deficits in spatial skills sometimes observed in blind children. For instance, lower performance in sound localization (Cappagli and Gori 2016; Vercillo et al. 2016) and auditory distance discrimination (Cappagli et al. 2017) have been reported in blind and visually impaired children from 6 to 17 years of age compared to sighted matched controls. It has been hypothesized that lower performance in spatial tasks is related to overall developmental delays (Ochaita and Huertas 1993) or specifically to delays in the development of locomotion (for a review, see Cuturi et al. 2016). Thus, it seems plausible to hypothesize that individuals who spend more time with locomotor activities show improved balance performance and a more typical gait pattern. Based on the observed link between balance performance and spatial cognitive functions, a positive correlation with navigation skills is additionally expected. These associations are predicted to be particularly expressed during childhood and adolescence, when both balance skills and spatial cognitive functions are subject of change.

In the present study, we assessed balance control, gait parameters, habitual physical activity, and navigation performance in a group of blind and visually impaired children and adolescents as well as in age-matched sighted controls. We hypothesized that physical activity is positively correlated with balance performance and gait parameters. Moreover, we predicted that physically active blind and visually impaired children and adolescents outperform their sedentary peers.

## Methods

### Participants

Fourteen blind and visually impaired children (mean age: 14.26 years, range 8–18, 8 females, 9 congenitally blind and 5 late blind) and 14 sighted children matched for age and gender with normal or corrected to normal vision (mean age: 14.00 years, range 8–18, 8 female) participated in the study. All children were recruited from the local communities of either Genoa (Italy) or Hamburg (Germany).

Blindness or visual impairment was due to retinopathy of prematurity (*n* = 3), congenital cataract and microphtalmia (*n* = 2), optic nerve glioma (*n* = 1), optic nerve atrophy (*n* = 1), tuberculous meningitis (*n* = 1), ocular albinism (*n* = 1), cones dystrophy (*n* = 1), homocystinuria (*n* = 1), Leber’s amaurosis (*n* = 1), retinoblastoma (*n* = 1), or unknown reasons (*n* = 1).

Of the blind and visually impaired individuals, two reported having no residual vision, and five reported rudimentary light and shadow perception. Visual acuity (decimal values) of the remaining seven participants was 0.1 (*n* = 4), 0.05 (*n* = 2), and 0.01 (*n* = 1).

The study was approved by the local ethical boards of the University of Hamburg and Genoa (Azienda Sanitaria Locale 3 Genovese) and was conducted in accordance with the ethical guidelines of the Declaration of Helsinki. Written informed consent was obtained from participants or from the parents of underage participants.

### Physical parameters

#### Postural sway

Postural sway was assessed with a force plate (Type 9260AA6, Kistler® Instrumente GmbH, Switzerland, sampling rate: 180 Hz) using the software BioWare (Kistler Instruments AG, version 4.0.1.2). Center of pressure displacement (CoP) data of the medial–lateral and anterior–posterior time series were collected during normal bipedal stance (both feet together) and during semi-tandem stance (the big toe of the dominant leg placed to the side of and against the heel of the other foot). During testing, participants were asked to direct their head straight ahead, with their hands placed on their hips. In closed eyes conditions, blind and sighted participants were blindfolded, and three trials per stance were run, each with a length of 30 s and an inter-trial rest of 30 s. Eyes open conditions were tested in sighted participants only, with three trials per stance with a length of 30 s each. The condition to start with (eyes open vs. eyes closed) was randomized for the sighted participants. To calculate the CoP sway area (in cm^2^) per trial, prediction ellipse areas (PEA) with 95% probability were fitted to the time series data per trial using MATLAB, version R2017b (for details on PEA see Duarte 2015; Schubert and Kirchner 2014). Technical problems during testing led to missing data of *n* = 1 blind and *n* = 2 visually impaired individuals. Data of the respective matched sighted individuals were removed for the analysis of postural sway. The mean sway area per stance and condition (eyes open/closed for the sighted) was used as dependent variable.

#### Single-leg stance time

Functional balance performance was assessed with barefoot single-leg stances (Springer et al. 2007) on hard ground. Participants were asked to place the hands on their hips, to lift their dominant foot, and to close their eyes if possible, with the head directed straight-ahead. Each trial had a length of 20 s, followed by 30 s of rest. Two trials were run. Trials were video-recorded, and two independent observers measured the time (in sec) the participant remained in the correct position. Trial time ended when participants touched the floor with their raised foot, rotated or moved their foot of the standing leg to maintain balance, removed their hands from the hips, or when sighted individuals opened their eyes. The mean time in sec across trials was used as dependent variable.

#### Gait parameters

Gait parameters were captured with a wireless inertial motion tracker (MTw sensors, Xsens Technologies B. V., Netherlands, sampling rate: 100 Hz) attached to the foot of the participants. During testing, participants walked up and down a hallway of approx. 25 m for 6 min and at their preferred walking speed wearing a blindfold. Sighted individuals were additionally tested with eyes open, the condition to start with was counterbalanced across participants. Two experimenters marked the ends of the hallway by constantly clicking their fingers. Once the participant reached the experimenter at the end of the hallway, he or she was asked to turn and walk back on the same way. Whenever a participant lost his path, he was guided back on the correct way. Before calculating the gait parameters, data of the first and the last 25 m as well as the first and the last 2.5 m of each 25 m bout were excluded. Furthermore, the kinematic time-series were visually checked. Areas with non-stationary data (e.g., when a participant stopped and was guided back on the correct way) were excluded from the following data analysis. The parameters gait velocity, stride length, stride time, minimum foot clearance and the variability of each parameter (standard deviation) were determined. The reliability of the system (inertial sensors and algorithms) has been verified (Hamacher et al. 2014). Furthermore, the largest Lyapunov exponent (LLE) as a measure of local dynamic gait stability (LDS) was calculated based on three-dimensional angular velocity data of the foot (Hamacher et al. 2015). To compute the LLE, we time-normalized the three-dimensional angular velocity data of 63 strides (minimum across participants and conditions) to 6300 samples. Using the delayed embedding approach, we chose the time delay (τ = 11) and the embedded dimension (dE = 15) based on the first minimal mutual information (Fraser and Swinney 1986) and the false nearest neighbor analysis (Kennel and Abarbanel 1992), respectively. Based on the resulting state-space, the LLE was calculated using Rosenstein and coworkers algorithm (Rosenstein et al. 1993). Higher LLE values are interpreted as lower LDS and vice versa. Gait parameters and LLE have been shown to depict reasonable construct and convergent validity (Bruijn et al. 2013). In this study, we report stride time variability (sd) and LDS, as these parameters have been shown to be related to physical activity and balance performance in healthy and fall-prone older adults (Hamacher et al. 2018; Hausdorff 2007), and have been used to characterize changes in gait due to diminished visual feedback (Hamacher et al. 2016).

#### Physical activity

The “Freiburger Questionnaire on Physical Activity” (Frey et al. 1999) was used to assess the overall self-reported weekly habitual physical activity. The questionnaire has been translated into Italian by one of the coauthors (G.C.) for usage in Italy. The questionnaire covers everyday basic physical activities such as taking the stairs and walking to school or work, leisure physical activities as well as physical exercise. For the present study, only questions on basic physical activity and physical exercise were included. The questionnaire has been answered with the help of the parents of underage participants. Activities were summarized for each category and are reported as minutes per week.

### Navigation performance

#### Triangle completion task

**Fig. 1:**
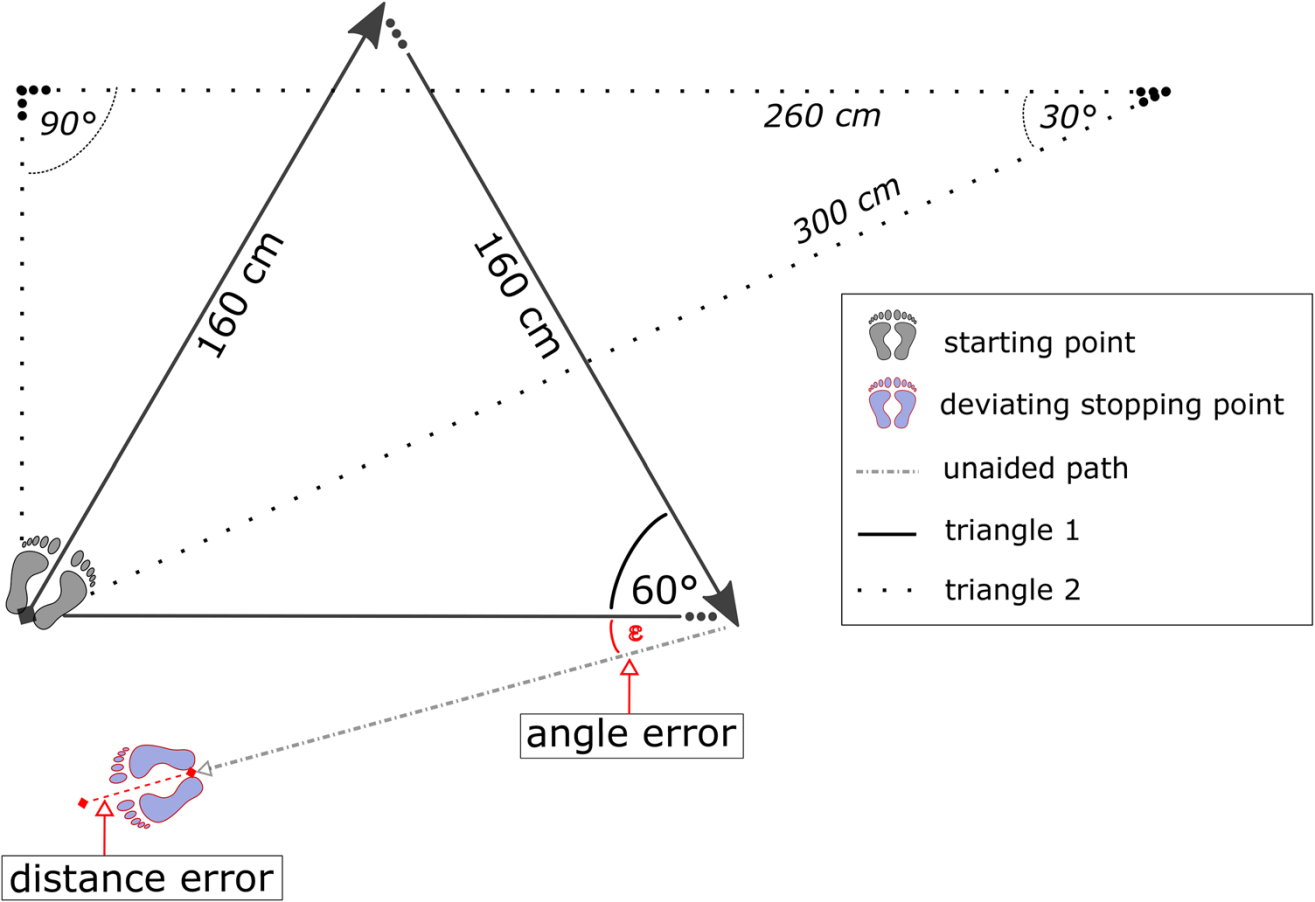
Triangles used in the path integration task (solid black lines: triangle 1, dotted black lines: triangle 2). The example depicts a deviation from the optimal path (solid lines) of triangle 1 performed in clockwise direction, with underestimated path length (red dashed line as distance error) and a turning error (red dotted line)

Navigation performance was assessed with a triangle completion task (Allen et al. 2004; Loomis et al. 2001). For this task, two triangles with route segment lengths between 150 and 300 cm (one equal-sided, one oblique triangle, resulting in target angles of 30°, 60° and 90°, respectively, see Fig. 1) were marked on the floor of an empty room. All participants were blindfolded and equipped with passive noise isolating headphones before entering the room. During testing, participants were guided along two segments of a triangle during which they touched the elbow of an experimenter. Upon reaching the end of the second path, the experimenter stepped aside and participants had to complete the triangle by turning and walking unaided to where they assumed the origin was. To calculate distance errors (in cm) and angle errors (in degrees), the stopping point of the participant was marked between the heels with adhesive tape on the floor after each trial. Four trials were run in a fixed order using the two triangles twice; once in clockwise and once in counterclockwise direction. The starting point was always the same. Between trials, participants were led on random walks within the room to avoid interference of previous trials. Participants did not receive feedback regarding their accuracy. Mean angle errors and mean (absolute) distance errors were used as dependent variables.

### Data analysis

Data were analyzed and visualized in R (v.3.6.2) (R Core Team 2020) using the R packages afex (v.0.25–1) (Singmann et al. 2018), tidyverse (v.1.2.1) (Wickham et al. 2019), and emmeans (v.1.4.5) (Lenth et al. 2020).

Data of blind and visually impaired individuals did not differ and were thus analyzed together as one group. Statistical analyses were performed using mixed factorial ANOVAs. Levene’s test was used to test for homogeneity of variance between groups. Heteroscedasticity-corrected covariance matrices using White-Huber adjustments are reported, in case of variance heterogeneity. Equations to assess differences in balance skills and navigation performance between blind and sighted individuals included the between-subjects factor Group (blind/visually impaired vs. sighted), and the within-subject factor Condition (bipedal vs. semi-tandem stance for the analysis of postural sway and basic activity vs. exercise for the analysis of physical activity). Eyes open data were available for postural stability and gait parameter for sighted participants only, resulting in unequal numbers of trials for the blind and visually impaired and the sighted. Therefore, two analyses were run: The first compared the blind with the sighted using eyes closed data. The second analysis used eyes closed and eyes open data of the sighted participants only to analyze the effect of vision. Statistical comparisons utilized Type III sums of squares; effects were considered significant at *p* < 0.05, post hoc comparisons used estimated marginal means with Tukeys correction for multiple comparisons. Standardized effect sizes utilized Cohen’s d with the respective 95% CI of the mean difference between Group (blind/visually impaired vs. sighted) or Condition (eyes closed vs. eyes open), complemented by unstandardized effect sizes as mean differences. Data visualization utilized violin plots per Group and Condition to show the distribution and density of the data.

To assess the relationship between physical parameters and navigation performance, separate Pearson correlations (two-sided) per group (blind/visually impaired and sighted) were performed using mean data of eyes closed conditions only.

## Results

### Balance performance

#### Postural sway

**Fig. 2:**
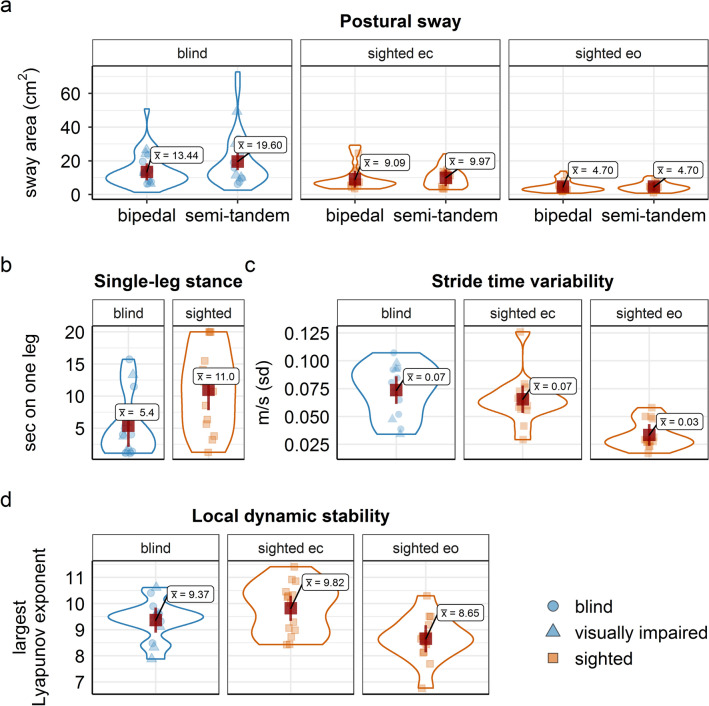
Violin plots for balance performance and gait parameters, separately for the blind and visually impaired (in blue) and for the sighted (in orange) group. Panel **a** postural sway per stance condition and for eyes open (eo)/eyes closed (ec) conditions in the sighted group; **b** single-leg stance time; **c** stride time variability; **d** local dynamic stability. Red squares represent the respective group mean; error bars depict 95% CI of the mean. Dots represent single-subject data

*Blind vs. sighted, eyes closed.* Postural sway was significantly larger for blind and visually impaired than for sighted individuals across stances when sighted were tested with closed eyes (*F* (1, 21) = 4.73, *p* = 0.041, mean difference = 6.99, *d* = 0.64, 95% CI [− 0.006, 1.29], see Fig. 2). Postural sway was larger in semi-tandem stance than in bipedal stance positions across groups (*F* (1, 21) = 7.04, *p* = 0.015, mean difference = 3.52, *d* = − 0.78, 95% CI [− 1.44, − 0.13]).

*Sighted, eyes open vs. eyes closed.* Postural sway was significantly larger in eyes closed than in eyes open conditions (*F* (1, 11) = 20.97, *p* < 0.001, mean difference = 4.98, *d* = 1.32, 95% CI [0.47, 2.18], with no significant difference between stance positions (*F* (1, 11) = 0.47, *p* = 0.505, mean difference = 0.44, *d* = -0.20, 95% CI [− 0.83, 0.43], see Fig. 2).

#### Single-leg stance time

*Blind vs. sighted, eyes closed.* Functional balance performance assessed with the single leg stance was significantly lower for blind and visually impaired than for sighted individuals (*F* (1, 25) = 6.32, *p* = 0.019, mean difference = − 5.6, *d* =− 0.97, 95% CI [− 1.81, − 0.13], see Fig. 2).

### Gait parameters

*Blind vs. sighted, eyes closed.* Blind and visually impaired and sighted participants did neither differ significantly in their stride time variability (*F* (1, 26) = 0.92, *p* = 0.345, mean difference = − 0.01, *d* =− 0.36, 95% CI [− 0.42, 1.15]) nor in local dynamic gait stability (*F* (1, 26) = 1.89, *p* = 0.181, mean difference = 0.45, *d* = 0.52, 95% CI [− 1.22, 0.27]), when sighted where tested with closed eyes.

*Sighted, eyes open vs. eyes closed.* Gait parameter of sighted individuals tested with eyes open differed significantly from test conditions with eyes closed: stride time variability decreased and local dynamic stability increased (lower LLE values) significantly in the eyes open condition as compared to the eyes closed condition (*F* (1, 13) = 27.00, *p* < 0.001, mean difference = − 1.17, *d* = 1.96, 95% CI [1.01, 2.92]) and (*F* (1, 13) = 39.34, *p* < 0.001, *d* = -2.37, 95% CI [1.11, 3.63]), see Fig. 2.

### Physical activity

**Fig. 3:**
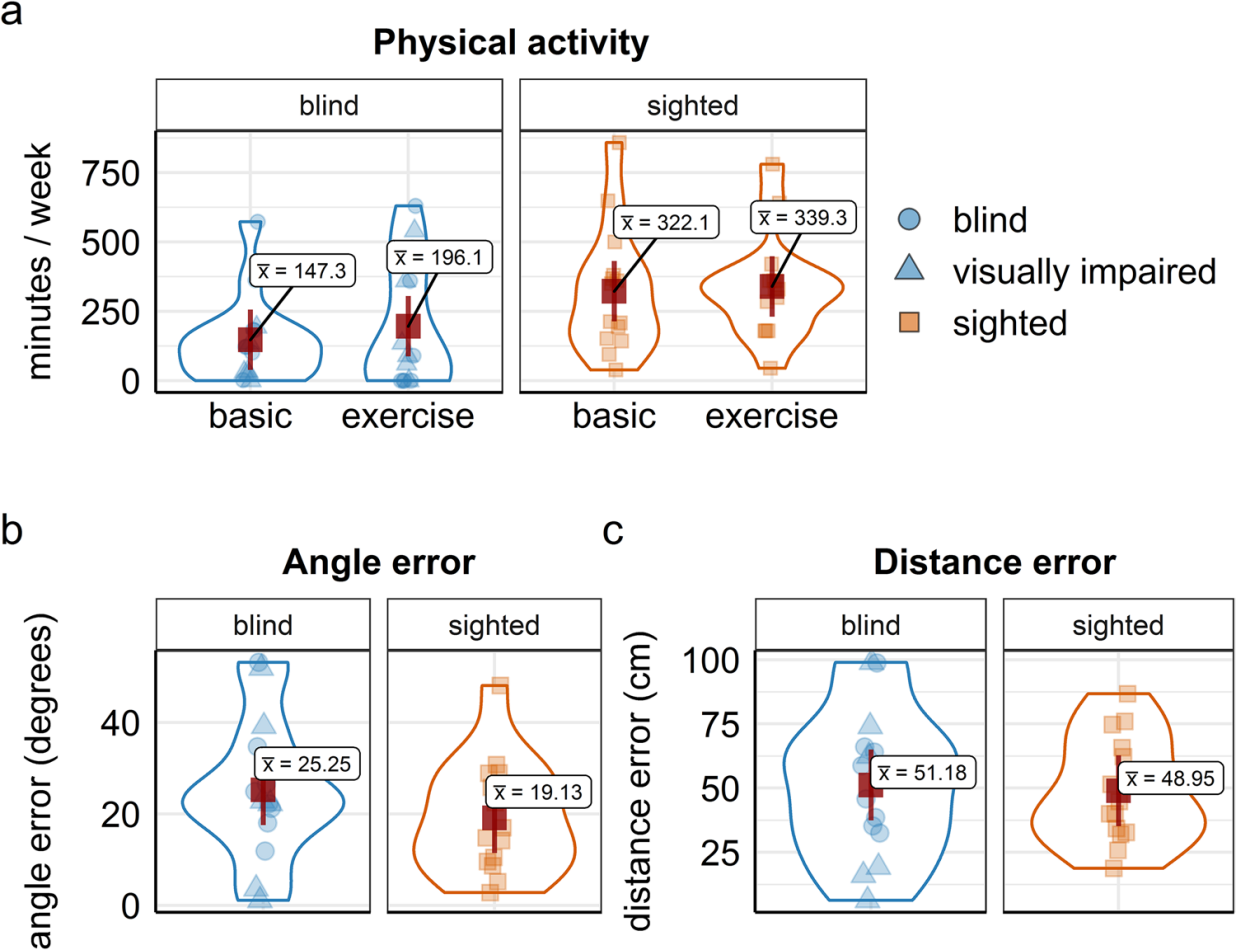
Violin plots for physical activity and navigation performance of blind and visually impaired (blue) and sighted (orange) individuals. Panel **a** self-reported weekly physical activity for basic everyday activities and physical exercise; **b** and **c** angle and distance errors assessed with the trial completion test. Red squares represent the respective group mean; error bars depict 95% CI of the mean, dots represent single-subject data

Self-reported physical activity was significantly lower in blind and visually impaired than in sighted individuals (*F* (1, 26) = 7.07, *p* = 0.013, mean difference = − 159, *d* =− 0.71, 95% CI [− 1.3, − 0.12]), with no significant difference between basic physical activities and physical exercise (*F* (1, 26) = 0.48, *p* = 0.494, *d* =− 0.18, 95% CI [− 0.72, 0.35]), see Fig. 3.

### Navigation performance

The blind and visually impaired group and the sighted group did not differ in their path integration performance, neither in their angle errors (*F* (1, 26) = 1.33, *p* = 0.258, mean difference = 6.12, *d* = 0.44, 95% CI [− 0.35, 1.22]) nor in their distance errors (*F* (1, 26) = 0.05, *p* = 0.816, mean difference = 2.23, *d* = 0.08, 95% CI [− 0.69, 0.87]), see Fig. 3).

### Relationships between physical activity, balance, gait, and navigation performance

#### Physical activity with balance, gait parameters, and navigation performance

**Fig. 4:**
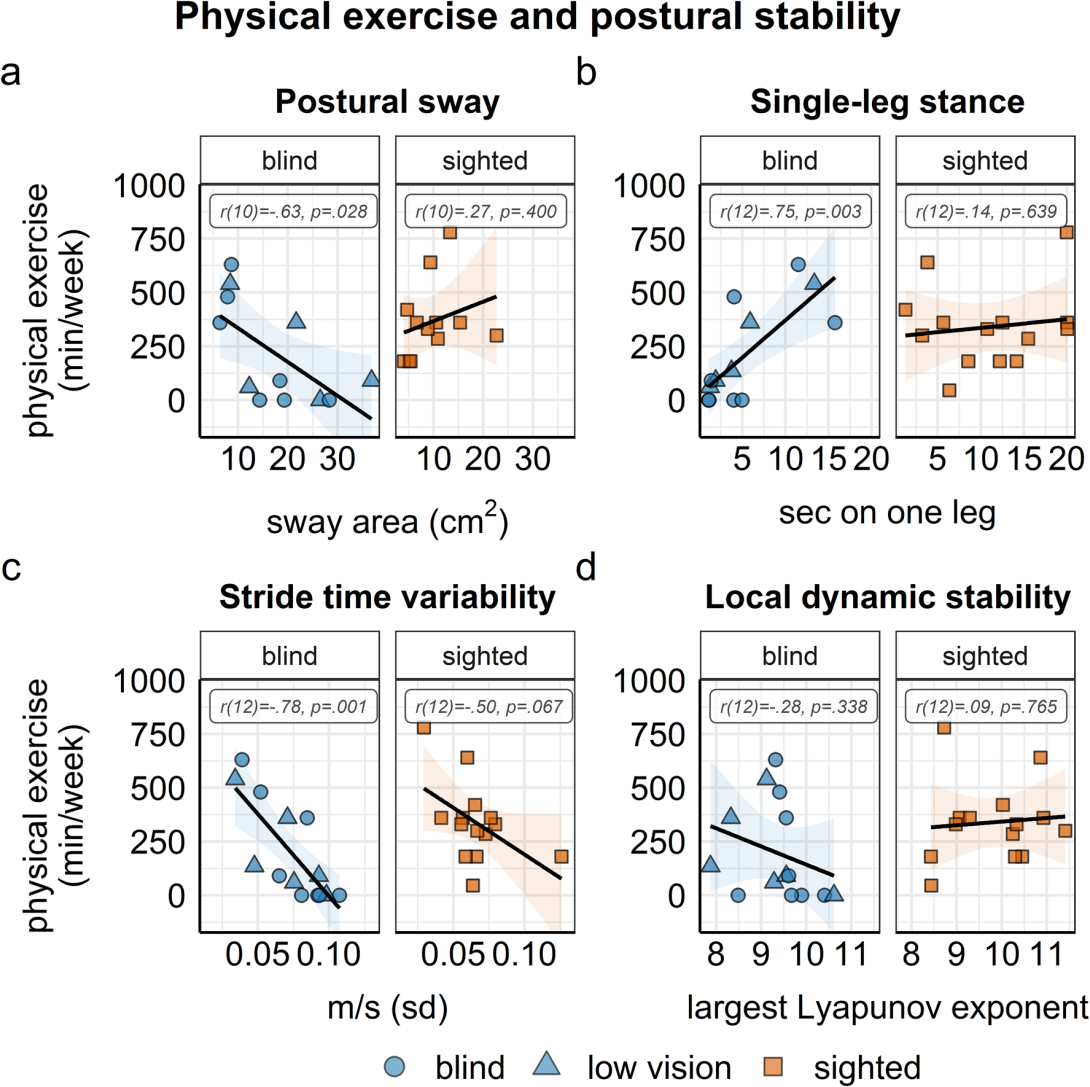
Correlations of physical exercise with balance performance (**a** and **b**) and gait parameters (**c** and **d**) within the blind (blue circles) and visually impaired (blue triangles) group and the sighted (orange rectangles) group. Error bands depict 95% CI, dots represent single-subject data

Correlational analyses revealed significant relationships between self-reported physical exercise and balance control, gait parameters, and navigation performance in the group of blind and visually impaired individuals. Higher levels of physical exercise were related to less postural sway (*r* (10) =  − 0.63, 95% CI [− 0.88, − 0.09], *p* = 0.028) and longer single-leg stance time (*r* (12) = 0.75, 95% CI [0.33, 0.92], *p* = 0.003) in the blind and visually impaired group, but not in the sighted group. Moreover, more physical exercise correlated significantly with lower stride time variability within the blind and visually impaired (*r* (12) =  − 0.78, 95% CI [− 0.93, − 0.4191], *p* = 0.001), but not within the sighted individuals (*r* (12) =  − 0.50, 95% CI [− 0.82, 0.40], *p* = 0.067, see Fig. 4). There were no significant correlations between basic everyday physical activity and balance and gait parameters, respectively (all *r* < 0.47, all *p* > 0.093, see supplementary material, Figure S3).

**Fig. 5:**
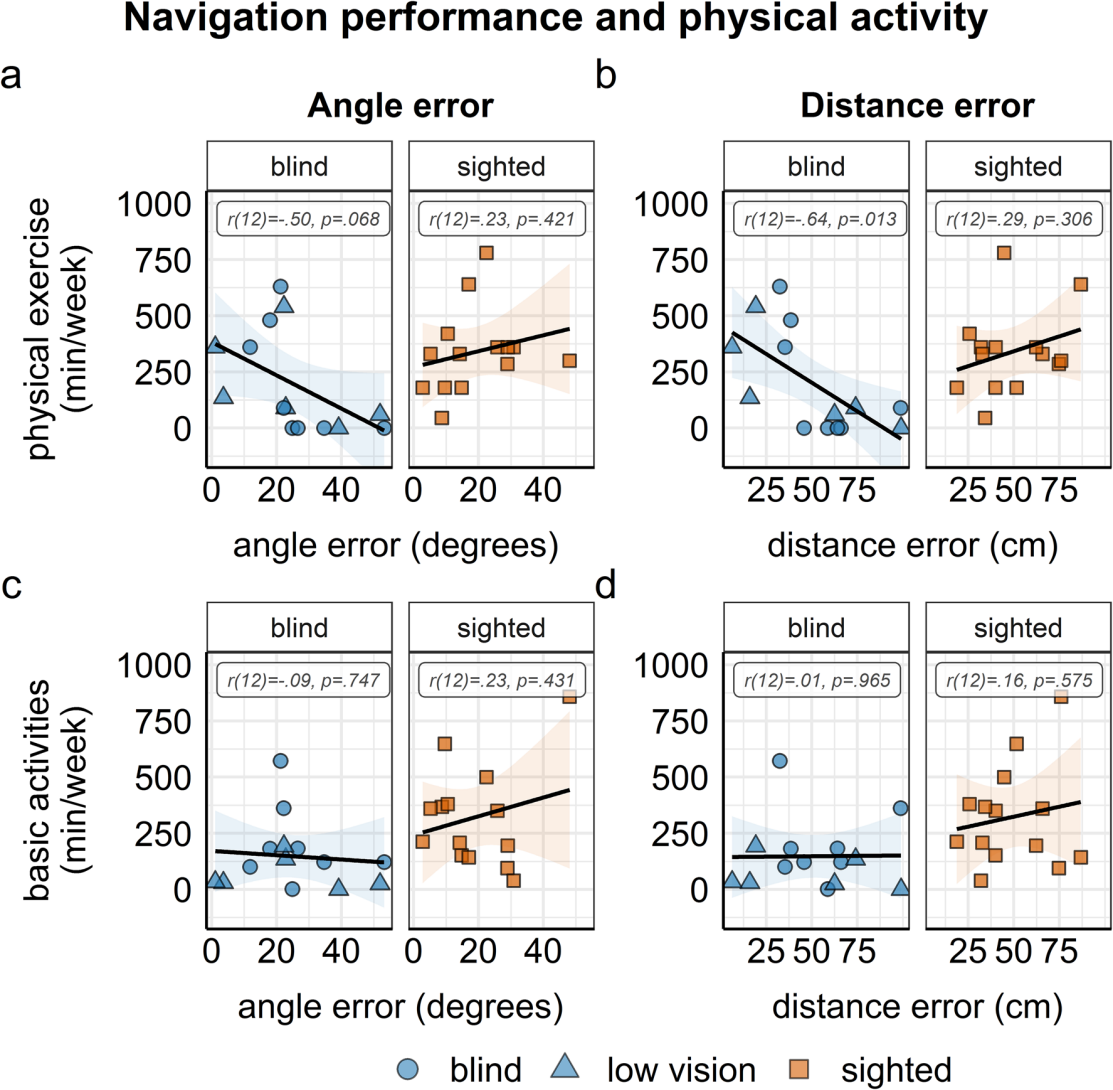
Correlations of navigation performance with physical exercise (**a** and **b**) and basic physical activities (**c** and **d**) within the blind (blue circles) and visually impaired (blue triangles) group and the sighted (orange rectangles) group. Error bands depict 95% CI, dots represent single-subject data

Navigation performance was related to physical exercise such that higher levels of physical exercise were significantly correlated with smaller distance errors (*r* (12) =  − 0.64, 95% CI [− 0.87, − 0.17], *p* = 0.013) and, marginally, with smaller angle errors in the triangle completion task (*r* (12) =  − 0.50, 95% CI [− 0.81, 0.04], *p* = 0.068) in the blind and visually impaired group, but not in the sighted group. There were no significant correlations between basic physical activity and navigation performance (all *r* < 0.23, all *p* > . 431), see Fig. 5.

#### Navigation performance with balance control and gait parameters

Correlations between navigation performance, balance control and gait parameters yielded the following pattern: Smaller angle errors were significantly related to less postural sway in the sighted group (*r* (10) = 0.83, 95% CI [0.48, 0.95], *p* < 0.001). In the blind group, the relationship between smaller distance errors and less postural sway as well as longer single-leg stance time, respectively, failed to reach significance (*r* (10) = 0.52, 95% CI [− 0.07, 0.84], *p* = 0.081, and *r* (12) =  − 0.54, 95% CI [− 0.84, 0.01], *p* = 0.056, see supplementary material, Figure S1). Navigation performance was linked to gait such that smaller distance errors were significantly correlated with less stride time variability (*r* (12) = 0.54, 95% CI [0.01, 0.81], *p* = 0.046) as well as with higher local dynamic stability (lower LLE values), *r* (12) = 0.63, 95% CI [0.14, 0.86], *p* = 0.017 in the blind and visually impaired group, but not in the sighted group (see supplementary material, Figure S2).

## Discussion

The present study investigated postural stability, gait parameters, physical activity, and navigation performance in blind and visually impaired as compared to sighted children and adolescents (8–18 years of age), and tested whether higher levels of physical activity are related to superior balance, gait, and navigation performance in blind participants. Postural sway was larger and single-leg stance time as well as physical activity were lower in blind and visually impaired participants than in blindfolded sighted participants. Blind and visually impaired individuals did not statistically differ from sighted individuals in stride time variability and local dynamic gait stability as well as navigation performance when the sighted were tested with closed eyes. Postural stability and gait parameters of sighted participants were significantly improved when visual input was available. Higher levels of physical exercise were related to enhanced balance performance, lower stride time variability, improved local dynamic gait stability, and superior navigation performance in blind and visually impaired children and adolescents.

*Postural stability and gait parameters.* The present results on balance performance of blind and visually impaired individuals are in line with studies reporting lower postural stability for blind or visually impaired than for sighted children (Müürsepp et al. 2018; Zipori et al. 2018) and sighted adults (Aydoğ et al. 2006; Campayo-Piernas et al. 2017; Giagazoglou et al. 2009; Ozdemir et al. 2013; Rogge et al. 2019; Schmid et al. 2007; Schwesig et al. 2011; Sobry et al. 2014), suggesting that postural stability is affected by the absence of visual cues, irrespective of the age of the individuals. The results have been interpreted as the absence of compensatory mechanisms in the blind, that is, no enhanced or superior use of non-visual input for balance control (Campayo-Piernas et al. 2017; Ozdemir et al. 2013; Schmid et al. 2007). Signs of superior non-visual self-motion perception in blind adults have been described, such as a faster decrease of postural sway when a light finger touch was allowed for postural stabilization (Schieppati et al. 2014), superior ankle proprioception, and enhanced vestibular roll tilt discrimination, compared to blindfolded adults (Moser et al. 2015; Ozdemir et al. 2013). Yet, these improvements did not result in enhanced balance performance of blind individuals compared to blindfolded sighted individuals. It has been suggested that behavioral compensation requires extensive practice of certain skills to show improvements (Kupers and Ptito 2014; Singh et al. 2018) and that blind individuals may have lacked practice due to overall lower engagement in physical exercise (Schmid et al. 2007). In line with this assumption, blind children as well as adults have been reported to adopt a more sedentary lifestyle (Augestad and Jiang 2015; Houwen et al. 2009; Longmuir and Bar-Or 2000; Müürsepp et al. 2018). In fact, earlier studies in blind and visually impaired individuals found that higher levels of habitual physical activity predicted superior balance performance as well as gait velocity (Aydoğ et al. 2006; da Silva et al. 2018; Müürsepp et al. 2018). Thus, it is reasonable to assume that physical exercise may foster balance skills and typical gait patterns. The data of the present study are in line with this hypothesis: Blind and visually impaired children and adolescents were less physically active than age-matched sighted controls, but those blind participants reporting more weekly physical exercise performed better in balance tasks than sedentary peers and moreover showed a lower stride time variability. While no causal relationship can be derived from a cross-sectional study design, we recently demonstrated in a longitudinal training study that balance training of no more than 12 weeks significantly improved balance performance of blind adults (Rogge et al. 2019), indicating that balance skills of blind individuals can be increased by specific practice. In the present study, balance performance of blind and visually impaired individuals was related to self-reported physical exercise, but not to everyday activities such as taking the stairs or walking to school. Thus, the observed correlations between physical exercise and balance control might result from demanding physical training including balance tasks.

*Navigation performance*. We observed no differences in path integration performance assessed with a triangle completion task between the group of blind and visually impaired and the group of blindfolded sighted individuals. This finding is in accord with earlier results showing no differences in path completion skills in congenitally and late blind adults compared to blindfolded sighted controls (Loomis et al. 1993). In contrast, other studies have reported both significantly worse and significantly better performance of congenitally or late blind individuals than of blindfolded controls in path completion tasks (Seemungal et al. 2007; Tinti et al. 2006). It has been speculated that the large variability of navigation performance within groups of blind participants as well as between different studies is partly explained by mobility skills as well as habitual physical activity of the blind (Loomis et al. 1993; Seemungal et al. 2007). The results of the present study are in line with this assumption: performance in the triangle completion task was significantly related to self-reported physical exercise, to local dynamic gait stability and, marginally, to postural stability within the group of blind and visually impaired children, suggesting that those blind individuals participating in more physical exercise have superior balance skills, show a more stable gait pattern, and perform better in navigation tasks than sedentary blind individuals. Path integration in the dark depends on vestibular information about translatory and rotatory accelerations of the head as well as proprioceptive information about self-motion (Glasauer et al. 2002). The vestibular system has been suggested to play a crucial role for spatial cognitive functions such as navigation, spatial memory and spatial learning (Hitier et al. 2014; Seemungal 2015). Vestibular pathways have been proposed to modulate neuroplasticity induced by physical exercise in brain regions associated with spatial cognitive functions and memory (Smith 2017). Moreover, vestibular self-motion processing and updating of one’s own position within the environment is extensively needed for physical exercise in the dark, such as blind soccer, judo, or athletic sports in the blind. In contrast, basic everyday activities were not related to navigation performance. It might thus be speculated that promoting physical exercise in blind individuals benefits not only postural stability and a stable gait pattern, but may enhance spatial navigation and orientation skills supporting individual mobility.

*Limitations.* Some limitations of the present study need to be considered. We included both blind and visually impaired children with an onset of visual deprivation at birth or later, leading to a heterogeneous sample with regard to the degree and duration of visual impairments. Previous studies on balance performance in blind individuals found no differences between congenitally and late blind individuals in postural stability (Rogge et al. 2019; Schmid et al. 2007) and gait parameters (da Silva et al. 2018). In our data, no systematic differences were observed between blind and visually impaired individuals in measures of balance control, gait, and levels of physical exercise. Similarly, navigation performance did neither differ between blind and visually impaired nor between congenitally and late blind participants.

In addition, blind and visually impaired children and adolescents were recruited from two different countries, which could lead to differences in mobility trainings, schools, and opportunities for physical activities for blind and visually impaired individuals. By looking at individual data, no systematic differences were observable between children from Italy and Germany, suggesting a robust relationship between physical exercise and postural stability, gait, and navigation performance in blind and visually impaired children.

Finally, the sample size of the present study was relatively small compared to similar studies in sighted children and adolescents, lowering statistical power and precluding the use of rigid corrections for multiplicity. Despite these limitations, our data revealed medium to strong associations between levels of physical activity and postural control, gait and navigation performance within the group of blind and visually impaired children and adolescents in the predicted direction. These results, thus, provide a starting point for further investigations.

*Conclusion.* Collectively, our findings suggest that physical activity may foster postural stability, gait, and navigation performance in blind and visually impaired children and adolescents. We speculate that this relationship is modulated by the vestibular system important for estimating self-motion. Our results suggest that rehabilitation efforts should include and promote a physically active lifestyle including exercises addressing postural stability to improve mobility and orientation skills in visually impaired individuals.

## Supplementary Information

Below is the link to the electronic supplementary material.

Supplementary file1 (PDF 4270 KB)

## Data Availability

The datasets generated for the present study are available from the corresponding author on reasonable request.
