# Supplementary material for: Balance, gait, and navigation performance are related to physical exercise in blind and visually impaired children and adolescents
Source: Exp Brain Res. 2021 Feb 7;239(4):1111–23. doi: 10.1007/s00221-021-06038-3 (PMC8068618; doi:10.1007/s00221-021-06038-3)
Supplement: Supplementary file 1 — Supplementary file1 (PDF 4270 KB) [file 221_2021_6038_MOESM1_ESM.pdf]

Supplementary material to:

Balance, gait, and navigation performance are related to physical exercise in blind and visually impaired children and adolescents. Experimental Brain Research.

Rogge, A.-K., Hamacher, D., Cappagli, G., Kuhne, L., Hötting, K., Zech, A., Gori, M., & Röder, B.

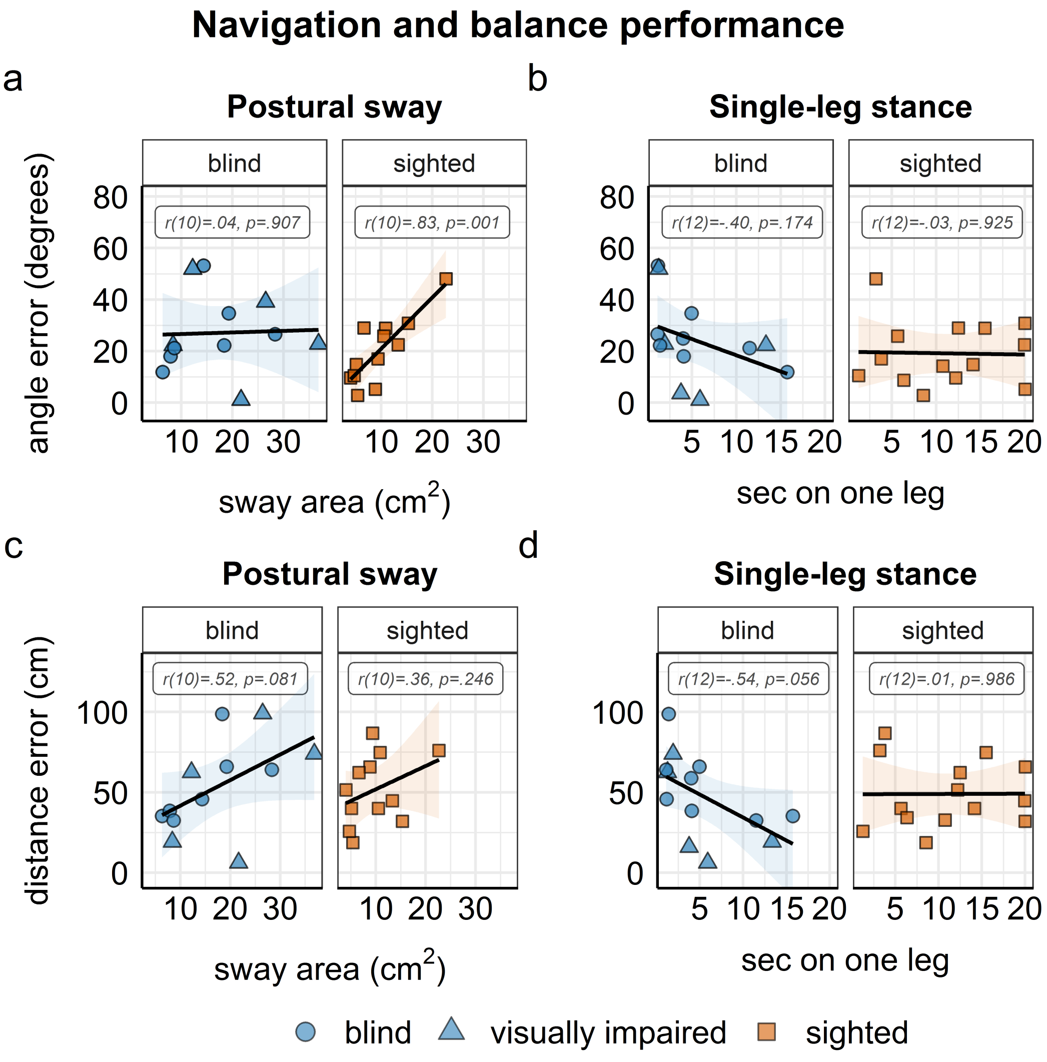

Figure S1 Correlations between navigation performance and balance performance within the blind (blue circles) and visually impaired (blue triangles) group and the sighted (orange rectangles) group, separately for angle errors (panel a and b) and for distance errors (panel c and d) assessed with the triangle completion task. Error bands depict 95% CI, dots represent single-subject data

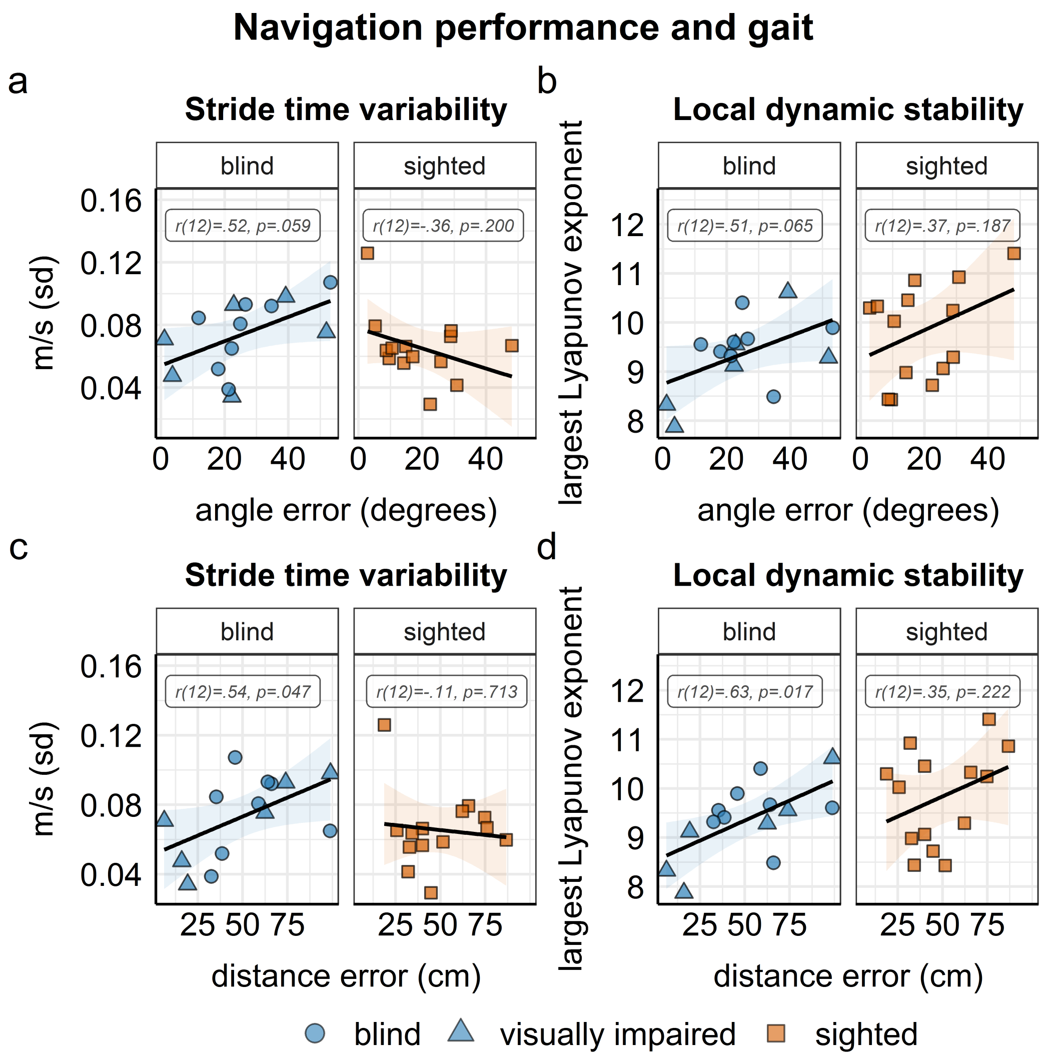

Figure S2 Correlations between navigation performance and gait parameters within the blind (blue circles) and visually impaired (blue triangles) and the sighted (orange rectangles) group, separately for angle error (panel a and b) and for distance errors (panel c and d). Error bands depict 95% CI, dots represent single-subject data.

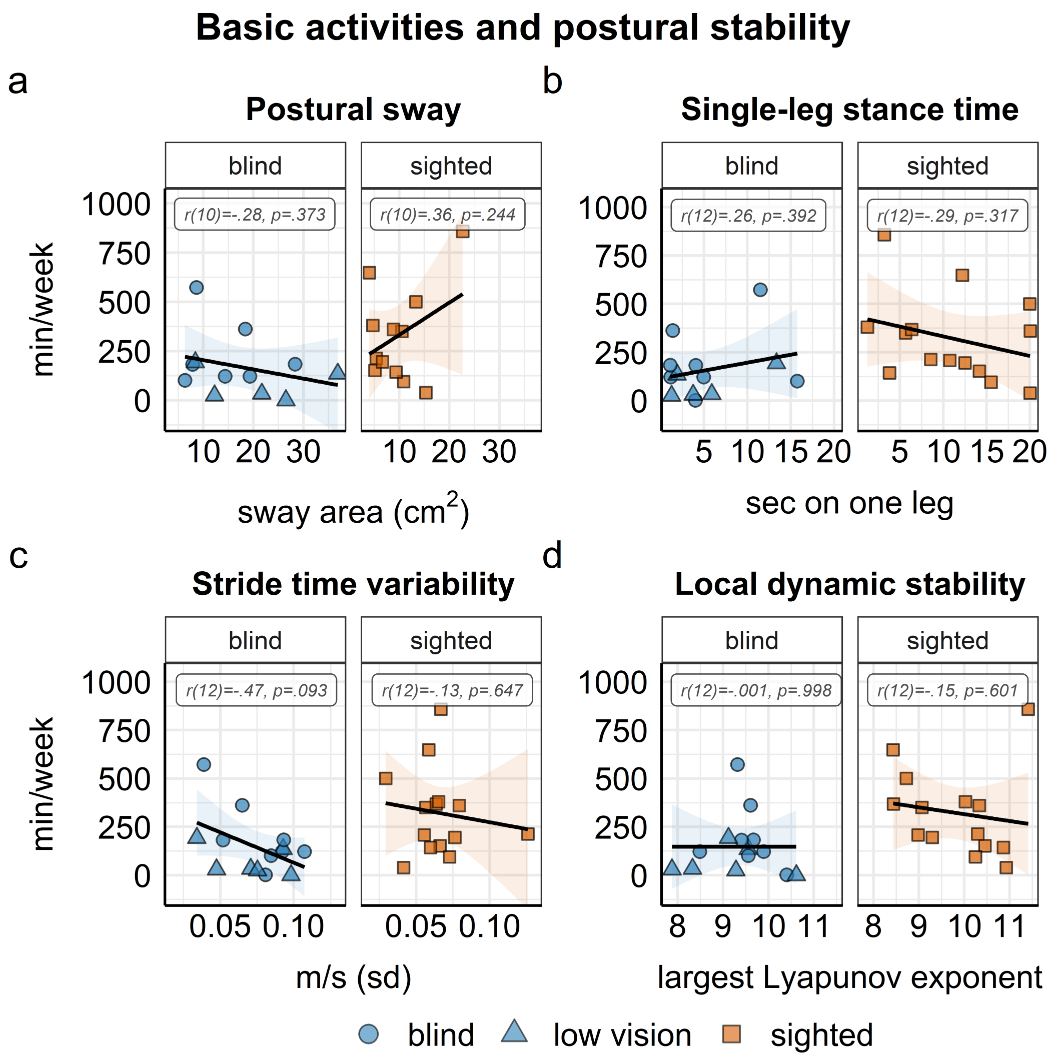

Figure S3 Correlations of everyday basic physical activities with balance performance (panel a and b) and gait parameters (panel c and d) within the blind (blue circles) and visually impaired (blue triangles) and the sighted (orange rectangles) group. Error bands depict 95% CI, dots represent single-subject data.
